# Department of Surgery

**Published:** 1894-02

**Authors:** 

**Affiliations:** Professor of Surgery in the Medical Department of the University of Texas, Galveston


### DEPARTMENT OF SURGERY.

EDITED BY PROF. J. E. THOMPSON, M. D.,
Professor of Surgery in the Medical Department of the University of Texas, Galveston.

GURLT (BERLIN). STATISTICS RELATIVE TO ANÆSTHESIA. (*Revue de Chirugie, Nov., 1893, page 953, Congres Allemand.*) A resumé of this subject, which the author has studied for two years, shows the following conclusions:

1. That pental is a dangerous anæsthetic, and ought not to be used (3 deaths in 597 cases).

2. Out of 133,729 cases where chloroform was given, 46 deaths occurred (i. e., 1 in 2,907).

3. Out of 14,646 cases where ether was administered, no deaths. Also no deaths after the A. C. E. mixture. Ether, therefore, appears to be the anæsthetic of choice.

In the discussion which followed, Koenig supported chloroform, which he had given in 7,000 cases without a death. He admitted that accidents were sufficiently common, but could be met by Mass's procedure of cardiac massage, which consists in energetic and rythmic blows over the heart (cardiac massage).

Küster preferred ether, and said that it was only contra-indicated in two sets of cases:

1. Where there is pulmonary mischief.
2. Where the operation is on the mouth or nose, and it cannot be efficiently administered.

---

F. KRAUSE (ALTONA). TRANSPLANTATION OF LARGE CUTANEOUS FLAPS WITHOUT PEDICLES. *Loc Cit.* The author prefers free flaps comprising the whole thickness of the skin, but no subcutaneous tissue. He has been very successful by this method in curing extensive ulcers of the face and leg. Out of more than one hundred flaps which he employed in twenty-four autoplastics, gangrene occurred in four cases only. He cuts them in an elongated form, from the inner surface of the arm or from the anterior surface of the thigh. They are dissected rapidly, and applied without sutures to the surface of the sore, which is previously vivified, allowing them to remain under the dressing for four days without being disturbed. It is necessary to take great precautions during the operation. Everything must be aseptic; the flaps washed with sublimate, and then with a solution of sea salt. Everything must be absolutely dry, and this refers as much to the hands and instruments as to the operative field.

---

HIRSCHBERG (FRANKFORT). TRANSPLANTATION OF FREE CUTANEOUS FLAPS CONTAINING SUBCUTANEOUS ADIPOSE TISSUE.—After combatting the opinion that all vital changes cease in a flap immediately the blood current ceases, he showed that circulatory currents continued for some time after removal of the flap, and furnished the first agglutinative material. It is evident that these currents will be more lively, the more rich the flap is in blood vessels, and Diffenbach has shown that free flaps adhere more easily if one causes them to become congested by previously tapping and rubbing them. He operated with complete success on four patients, in the following manner:

After the application of Esmarch's bandage to the upper extremity, he struck for two or three minutes the upper and inner part of the arm with a rubber hammer. Then he cut a quadrilateral flap of slightly larger dimensions than the loss of substance, detached it from the subjacent aponeurosis on three sides, leaving it attached by a pedicle turned towards the wrist. The ulcer was then vivified, and all bleeding stopped. He then removed Esmarch's bandage, and in a few minutes the flap became red and turgid. Its attachments were cut with strong scissors, and the flap was carefully applied to the loss of substance. He used gauze, or lint, as a dressing.

---

BREMANN (HALLE). TREATMENT OF GUNSHOT WOUNDS OF THE ABDOMEN.—A resumé of his conclusions, based on observation of cases, is:

1. In all gunshot wounds of the abdomen, accompanied immediately with signs of a lesion of the stomach or intestine, or of a severe internal hemorrhage, laparotomy should be done at once.

2. Laparotomy is also urgently indicated in cases where the seat and the direction of the projectile admit the possibility of a wound in the stomach or intestine.

In support of this statement, he quotes MacCormic's statistics, that ninety-nine per cent of patients not operated on will die.

---

KORTE. "INTESTINAL OBSTRUCTION BY BILIARY CALCULI." —The author has operated on three patients with the above condition, with two cures and one death. In all the cases the stones, although they were not very large, were so fixed that they could not be pushed forward or backward. Two were situated in the ileum, and one in the sigmoid flexure. The clinical symptoms were those of a very grave strangulation.

Medical treatment produced no good result. The calculus was easily found, the intestine was opened by a longitudinal incision, the stone removed, and the wound sutured.

---

BORCK (ROSTOCK). "DISLOCATION OF THE SEMILUNAR CARTILAGES OF THE KNEE."—He reports the case of a man who sustained a severe twist of the right knee three years ago. After a time he regained movement in the joint, but sometime afterwards fell from his horse on the knee. He suffered with intense pain, hæmarthrosis, and signs of a foreign body.

An incision was made over the inner side of the joint, and the foreign body was shown to be the anterior third of the semilunar cartilage, which had preserved only its attachment to the anterior part of the spine of the tibia. The meniscus was removed, and the joint closed. Cure was complete in thirty days.

